# Syntheses and Patterns of Changes in Structural Parameters of the New Quaternary Tellurides Eu*RE*CuTe_3_ (*RE* = Ho, Tm, and Sc): Experiment and Theory

**DOI:** 10.3390/ma17143378

**Published:** 2024-07-09

**Authors:** Anna V. Ruseikina, Maxim V. Grigoriev, Ralf J. C. Locke, Vladimir A. Chernyshev, Thomas Schleid

**Affiliations:** 1 Laboratory of Theory and Optimization of Chemical and Technological Processes, University of Tyumen, 625003 Tyumen, Russia; ma.v.grigorev@utmn.ru; 2 Institute for Inorganic Chemistry, University of Stuttgart, D-70569 Stuttgart, Germany; ralf.locke@iac.uni-stuttgart.de; 3 Institute of Natural Sciences and Mathematics, Ural Federal University named after the First President of Russia B.N. Yeltsin, 620002 Ekaterinburg, Russia; vchern@inbox.ru

**Keywords:** quaternary europium copper tellurides, syntheses, crystal structure, DFT calculations, rare-earth metals

## Abstract

The layered orthorhombic quaternary tellurides Eu*RE*CuTe_3_ (*RE* = Ho, Tm, Sc) with *Cmcm* symmetry were first synthesized. Single crystals of the compounds up to 500 μm in size were obtained by the halide-flux method at 1120 K from elements taken in a ratio of Eu/*RE*/Cu/Te = 1:1:1:3. In the series of compounds, the changes in lattice parameters were in the ranges *a* = 4.3129(3)–4.2341(3) Å, *b* = 14.3150(9)–14.1562(9) Å, *c* = 11.2312(7)–10.8698(7) Å, *V* = 693.40(8)–651.52(7) Å^3^. In the structures, the cations Eu^2+^, *RE*^3+^ (*RE* = Ho, Tm, Sc), and Cu^+^ occupied independent crystallographic positions. The structures were built with distorted copper tetrahedra forming infinite chains [CuTe_4_]^7−^ and octahedra [*RE*Te_6_]^9−^ forming two-dimensional layers along the *a-*axis. These coordination polyhedra formed parallel two-dimensional layers CuRETe32−∞2. Between the layers, along the *a*-axis, chains of europium trigonal prisms [EuTe_6_]^10−^ were located. Regularities in the variation of structural parameters and the degree of distortion of coordination polyhedra depending on the ionic radius of the rare-earth metal in the compounds Eu*RE*Cu*Ch*_3_ (*RE* = Ho, Er, Tm, Lu, Sc; *Ch* = S, Se, Te) were established. It is shown that with a decrease in the ionic radius *r_i_*(*RE*^3+^) in the compounds Eu*RE*CuTe_3_, the unit-cell volume, bond length *d*(*RE*–Te), distortion degree [CuTe_4_]^7−^, and crystallographic compression of layers [*RE*CuTe_3_]^2−^ decreased. The distortion degree of tetrahedral polyhedra [Cu*Ch*_4_]^7−^, as well as the structural parameters in europium rare-earth copper tellurides Eu*RE*CuTe_3_, were higher than in isostructural quaternary chalcogenides. Ab initio calculations of the crystalline structure, phonon spectrum, and elastic properties of compounds Eu*RE*CuTe_3_ (*RE* = Ho, Tm, and Sc) ere conducted. The types and wave numbers of fundamental modes were determined, and the involvement of ions in IR and Raman modes was assessed. The calculated data of the crystal structure correlated well with the experimental results.

## 1. Introduction

Studying four-component tellurides opens up new perspectives for creating materials with unique properties and potential applications in various fields, including electronics, optics, and energy [1,2,3,4,5,6]. Understanding the influence of structural features on the properties of chalcogenide materials allows for the modification of the band gap width, electrical, and optical properties of the materials [7,8,9,10,11,12,13,14,15]. The presence of a large variety of coordination polyhedra and different ways of connecting them in tellurides creates conditions for the formation of layered or tunnel structures [6,9]. Layered tellurides of the space group *Cmcm* demonstrate promising efficiency in solar energy conversion and are considered as prospective absorbers in solar cell structures, as well as thermoelectric materials [1,2,3,4]. It is assumed that in compounds of the space group *Cmcm*, such as *MRE*CuTe_3_ (*M* = *d*-element) with the structure type of KZrCuS_3_, there is a covalently bonded sublattice [*RE*CuTe_3_]^2−^, leading to improved electrical transport properties, and the *M*^2+^ cations induce low lattice thermal conductivity [1,2]. Tellurides of the space group *Cmcm* contain non-centrosymmetric tetrahedral coordination polyhedra [16], allowing them to be used in nonlinear optical devices as photon media [17,18]. In the Eu*RE*Cu*Ch*_3_ structures, a tetrahedral structural motif is clearly visible in the form of one-dimensional chains of distorted [Cu*Ch*_3_]^5−^ [18,19,20,21,22,23,24,25,26] tetrahedra. Compounds Ba*RE*Cu*Ch*_3_ (*Ch* = chalcogen) are used as hole transport materials in solar cells, significantly increasing the efficiency of the solar cell (up to 45%) compared to a cell without this chalcogenide [1,27,28]. It was previously established that replacing the alkaline earth *M*^2+^ ions in the quaternary chalcogenides *MRE*Cu*Ch*_3_ with the Eu^2+^ cation makes it possible to narrow the band gap of the compound due to the presence of the 4f–5d transition in the Eu^2+^ ion [8]. Also, an increase in the chalcogen radius in the series *r_i_*(S^2−^) > *r_i_*(Se^2−^) > *r_i_*(Te^2−^) reduces the value of the band gap [7]. Thus, it is expected that the synthesis of Eu*RE*CuTe_3_ compounds will make it possible in the future to obtain semiconductor materials with the bandgap value required for photovoltaic materials and use them as a photoanode in multilayer solar cells, improving charge separation as well as reducing the recombination of electrons and holes at the interface of the photoanode and electrolyte.

**Figure 1:**
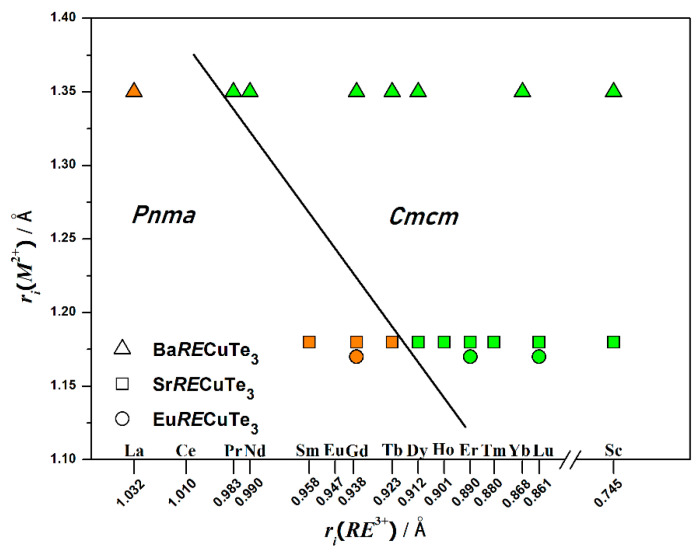
Structure field diagram of *MRE*CuTe_3_ tellurides with *M* = Ba [1,16,17,30,31], Sr [1,12,16], Eu [19,20]. Description: color background corresponds to a defined structure type (space group *Pnma*—orange (Eu_2_CuS_3_); space group *Cmcm*—green (KZrCuS_3_)). The black line delimits the regions of existence of the space groups *Pnma* and *Cmcm*.

Experimental and theoretical studies of the sulfide series of europium compounds Eu*RE*CuS_3_ were carried out for quite a long time from 1986 to 2021 [8,22,23,24,29], and similar studies of the selenide series Eu*RE*CuSe_3_ were carried out from 2022 to 2024 [18,25,26]. Research has shown that the compounds are p-type semiconductors, low-temperature ferro- and ferrimagnets, with a negative magnetization effect, high-temperature stability in an inert atmosphere, and polymorphism [8,16,18,22,23,24,25,26,29]. A decrease in incongruent melting temperatures and band gaps with changes in chalcogen has been established [8,24,26]. From the Eu*RE*CuTe_3_ series of europium tellurides, only three have been obtained to date: EuGdCuTe_3_ [20], EuErCuTe_3_ [19], and EuLuCuTe_3_ [20] (Figure 1). However, quaternary tellurides with alkaline earth elements Ba*RE*CuTe_3_ (*RE* = Pr–Yb, Sc) [1,16,17,30,31] and Sr*RE*CuTe_3_ (*RE* = Dy–Lu, Sc) [1,12,16] are described in detail in the literature. The smaller the ionic radius of the *M*^2+^ cation in the compounds *MRE*CuTe_3_, the fewer compounds will crystallize in the space group *Cmcm* [12]. The Eu^2+^ cation has a smaller ionic radius than the Sr^2+^ and Ba^2+^ cations [32]. It can be assumed that compounds Eu*RE*CuTe_3_ (*RE* = Ho–Lu and Sc) will crystallize in the space group *Cmcm*.

For compounds EuTmCuTe_3_ and EuScCuTe_3_, the structure type of NaCuTiS_3_ has been predicted [2], crystallizing in the space group *Pnma* [33]. According to DFT calculations using the PBE functional in this space group, the band gap widths of these compounds are 0.59 and 0.40 eV, respectively [2]. The structure of the orthorhombic crystal NaCuTiS_3_ is characterized by distorted copper tetrahedra and titanium octahedra forming two-dimensional layers [CuTiS_3_]^−^ along the *c*-axis [33]. Single-capped trigonal prisms of sodium are located between the layers. However, previously obtained scandium and thulium quaternary chalcogenides *M*R*E*Cu*Ch*_3_ (*M* = Eu [8,18,22,24,25], Sr [8,12,34,35,36], Ba [1,37,38,39]; *RE* = Sc, Tm; *Ch* = S, Se, Te) crystallize in the space group *Cmcm* with the KZrCuS_3_-type structure. It is expected that compounds EuScCuTe_3_ and EuTmCuTe_3_ will crystallize in the same structure type and space group as compounds with isostructural composition [22,24,25,35,36,37,38,39].

There are no literature data on attempts to synthesize and establish the crystal structure of tellurides Eu*RE*CuTe_3_ (*RE* = Ho, Tm, Yb, Sc). In this work, we first describe the single-crystal synthesis of Eu*RE*CuTe_3_ (*RE* = Ho, Tm, and Sc), investigating their crystal structure both experimentally and using theoretical methods.

## 2. Experimental

### 2.1. Materials

Ho (99.9%), Tm (99.9%), Yb (99.9%), Sc (99.9%), Eu (99.99%), Te (99.9%), Ar (99.99%), C_2_H_2_ (99.9%), and CsI (99.9%) were purchased from ChemPur (Karlsruhe, Germany). Cu (99.999%) was obtained from Aldrich (Milwaukee, WI, USA).

### 2.2. Synthesis

**Figure 2:**
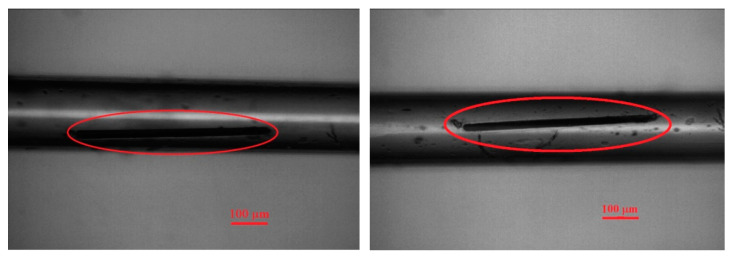
Photograph of EuHoCuTe_3_ (**left**) and EuScCuTe_3_ (**right**) crystals placed in a capillary for X-ray diffraction analysis.

Single crystals of Eu*RE*CuTe_3_ (*RE* = Ho, Tm, Sc) were synthesized from elements taken in a ratio of Cu/Eu/*RE*/Te = 1:1:1:3 with the addition of cesium iodide as a flux. Due to the rapid oxidizability of rare-earth metals by components of the air (oxygen, carbon dioxide, water vapor) at room temperature, the starting components were weighed in an inert atmosphere using a glovebox. A layer of amorphous carbon obtained by pyrolysis of acetylene was pre-applied to the inner walls of the silica ampoules. After filling the ampoules with the starting components, they were tightly sealed with a quick-release seal, removed from the glove box, connected to a vacuum pump, evacuated to a residual pressure of 2 × 10^−3^ mbar, sealed, and heated in a muffle furnace. The temperature in the furnace was raised from room temperature to 1120 K over 30 h with subsequent isothermal holding for 96 h. Cooling was carried out to 570 K over 140 h and to 300 K over 3 h. Residual flux from the sample was removed with demineralized water. The products were black needle-like crystals of Eu*RE*CuTe_3_ (*RE* = Ho, Tm, Sc) up to 500 µm in size (Figure 2). The obtained crystals were suitable for single crystal X-ray diffraction analysis. Unfortunately, it was not possible to obtain high-quality powder diffraction patterns, as copper compounds strongly absorb molybdenum radiation. The yield of tellurides was 10–15%, which did not allow for the experimental determination of the optical properties of the samples. Unfortunately, despite numerous attempts, we were unable to obtain the compound EuYbCuTe_3_ using the flux method. The samples after synthesis contained single crystals of Cu_0.37_YbTe_2_, EuTe, and YbTe. The phase of EuYbCuTe_3_ was not found in the samples, possibly due to the weak oxidizing ability of tellurium interfering with the Yb^0^–3e^−^→Yb^3+^ redox reaction.

### 2.3. X-ray Diffraction Analysis

The intensities from a single crystal of the Eu*RE*CuTe_3_ (*RE* = Ho, Tm, Sc) were collected at 293(2) K using a SMART APEX II single-crystal diffractometer (Bruker AXS, Billerica, MA, USA) equipped with a CCD-detector, graphite monochromator, and Mo-*K*_α_ radiation source. The parameters of the unit cell were determined and refined for a set of 11,880 reflections. The parameters of the unit cell correspond to the orthorhombic crystal system. The space group *Cmcm* was determined from the statistical analysis of all intensities. Absorption corrections were applied using the SADABS program. The crystal structure was solved by direct methods using the SHELXS program and refined in an anisotropic approximation using the SHELXL program [40]. Structural investigations for the presence of missing symmetry elements were conducted using the PLATON program [41]. The crystallographic data are deposited in Cambridge Crystallographic Data Centre. The data can be downloaded from the site www.ccdc.cam.ac.uk/data_request/cif (accessed on 25 February 2024).

### 2.4. DFT Calculations

Calculations were performed at the theoretical level DFT by using hybrid functional B3LYP [42] that take into account non-local exchange at Hartree–Fock formalism. This approach is suitable for to compounds with ionic and covalent chemical bonds. We used CRYSTAL17 code [43]. The program is designed for modeling periodic compounds within the framework of the MO-LCAO approach. Stuttgart pseudopotentials ECP*n*MWB for Eu^2+^ and *RE*^3+^ cations were used [44]. Here, *n* is the number of electrons that replaces this pseudopotential. For Eu^2+^, *n* was equal Z–10, and for RE^3+^, *n* was equal Z–11 (Z is atomic number). For outer shells 5*s*^2^5*p*^6^ of rare-earth metal ions, which were involved in chemical bonds, attached basis sets of TZVP type were used [44]. For scandium and copper, we used all-electron basis sets «Sc_pob_TZVP_2012» and «Cu_86-4111(41D)G_doll_2000», available on the website of program CRYSTAL [43]. For tellurium, we used an all-electron basis set [45] also. Gaussian-type orbitals with exponents lower than 0.1 were cancelled from the basis sets. Self-consistent field was calculated with tolerance 10^−9^ a.u. Monkhorst-Pack grid used was 8 × 8 × 8 k-points.

Optimization of the crystal structure was performed at first. After that, elastic constants and phonon spectrum were calculated for optimized crystal structure.

## 3. Results

### 3.1. Crystal Structures of the EuRECuTe_3_ (RE = Ho, Tm, Sc)

In the series of europium tellurides Eu*RE*CuTe_3_, only three orthorhombic compounds Eu*RE*CuTe_3_ (*RE* = Gd [20], Er [19], Lu [20]) have been previously obtained using the halide flux method. The compound EuGdCuTe_3_ [20] crystallizes in the space group *Pnma* in the structure type of Eu_2_CuS_3_, while the compounds EuErCuTe_3_ [19] and EuLuCuTe_3_ [20] crystallize in the space group *Cmcm* in the structure type of KZrCuS_3_. Single-crystal X-ray diffraction analysis revealed that the compounds Eu*RE*CuTe_3_ (*RE* = Ho, Tm, Sc) are isostructural to Eu*RE*CuTe_3_ (*RE* = Er [19], Lu [20]). Crystallographic data and data collection conditions are presented in Table 1.

**Figure 3:**
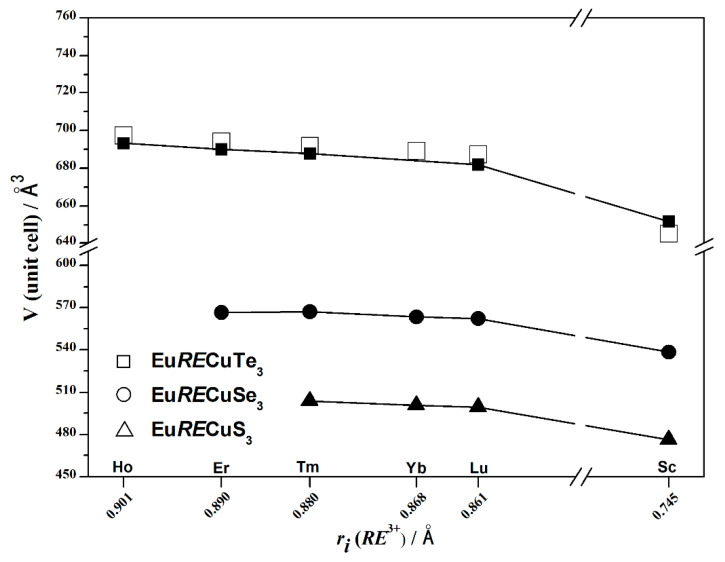
Relationship between the experimental (shaded shapes) and calculated (unshaded shapes) values of the unit-cell volume as a function of the ionic radius of the rare-earth metal cation in the series of the compounds Eu*RE*CuTe_3_ (*RE* = Ho [this work], Er [19], Tm [this work], Yb [this work], Lu [20], Sc [this work]), Eu*RE*CuSe_3_ (*RE* = Er [26], Tm–Lu [25], Sc [18]), and Eu*RE*CuS_3_ (*RE* = Tm–Lu [22], Sc [8]).

**Figure 4:**
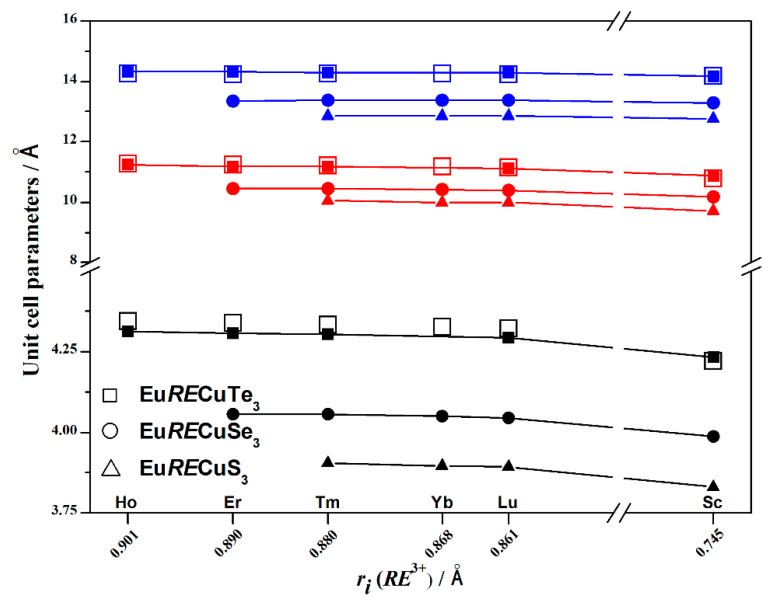
Relationship between the unit-cell parameters (*a*: black, *b*: blue, *c:* red) and the ionic radius of the rare-earth metal cation in the series of compounds Eu*RE*CuTe_3_ (*RE* = Ho [this work], Er [19], Tm [this work], Yb [this work], Lu [20], Sc [this work]), Eu*RE*CuSe_3_ (*RE* = Er [26], Tm–Lu [25], Sc [18]), and Eu*RE*CuS_3_ (*RE* = Tm–Lu [22], Sc [8]).

The atomic coordinates, thermal displacement parameters, bond lengths, and valence angles are presented in Tables S1–S3 of the Supplementary Material. The lattice parameters obtained from DFT calculations of Eu*RE*CuTe_3_ (*RE* = Ho, Tm, Lu, Sc; Table S4) were in good agreement with the experimentally determined values (Table 1). The compound EuYbCuTe_3_ was unable to be obtained using the halide flux method, but since Yb^3+^ lies between Tm^3+^ and Lu^3+^, whose quaternary tellurides crystallize in the structure type of KZrCuS_3_, if EuYbCuTe_3_ is experimentally obtained by another method, it will also crystallize in this structure type. Based on this assumption, DFT calculations of the crystal structure, phonon spectrum, and elastic properties of EuYbCuTe_3_ were carried out in this study. The calculated values of the electronic parameters and volume were in line with the general trend of their variations (Figure 3 and Figure 4).

In the series of quaternary tellurides Eu*RE*CuTe_3_, where *RE* = Ho–Lu and Sc, the unit-cell volume decreases from 693.40 to 651.52 Å^3^ (Table 1, Figure 3), and the unit-cell parameters (*a* = 4.3129–4.2341 Å, *b* = 14.3150–14.1562 Å, *c* = 11.2312–10.8698 Å) decreased, which is consistent with the change in structural parameters as the ionic radius of *RE*^3+^ decreases in the series of Eu*RE*Cu*Ch*_3_ chalcogenides (*Ch* = S [22,24], Se [25]), crystallizing in the space group *Cmcm* (Figure 3 and Figure 4). When changing the chalcogenide in the series Te^2−^ → Se^2−^ → S^2−^, the anion radii decrease by 10% and 7% (*r_i_*(Te^2−^) = 2.21 Å, *r_i_*(Se^2−^) = 1.98 Å, *r_i_*(S^2−^) = 1.84 Å [32]), respectively. As a result, the unit-cell volumes changed by approximately 18% and 12% (Figure 3), and the unit-cell parameters changed by 6% and 4%, respectively (Figure 4).

**Figure 5:**
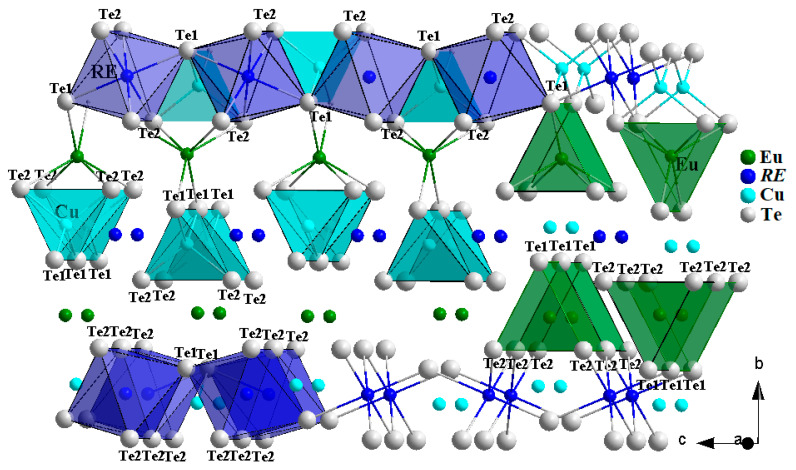
Projection of the crystal structure of the Eu*RE*CuTe_3_ representatives in the space group *Cmcm*.

**Figure 6:**
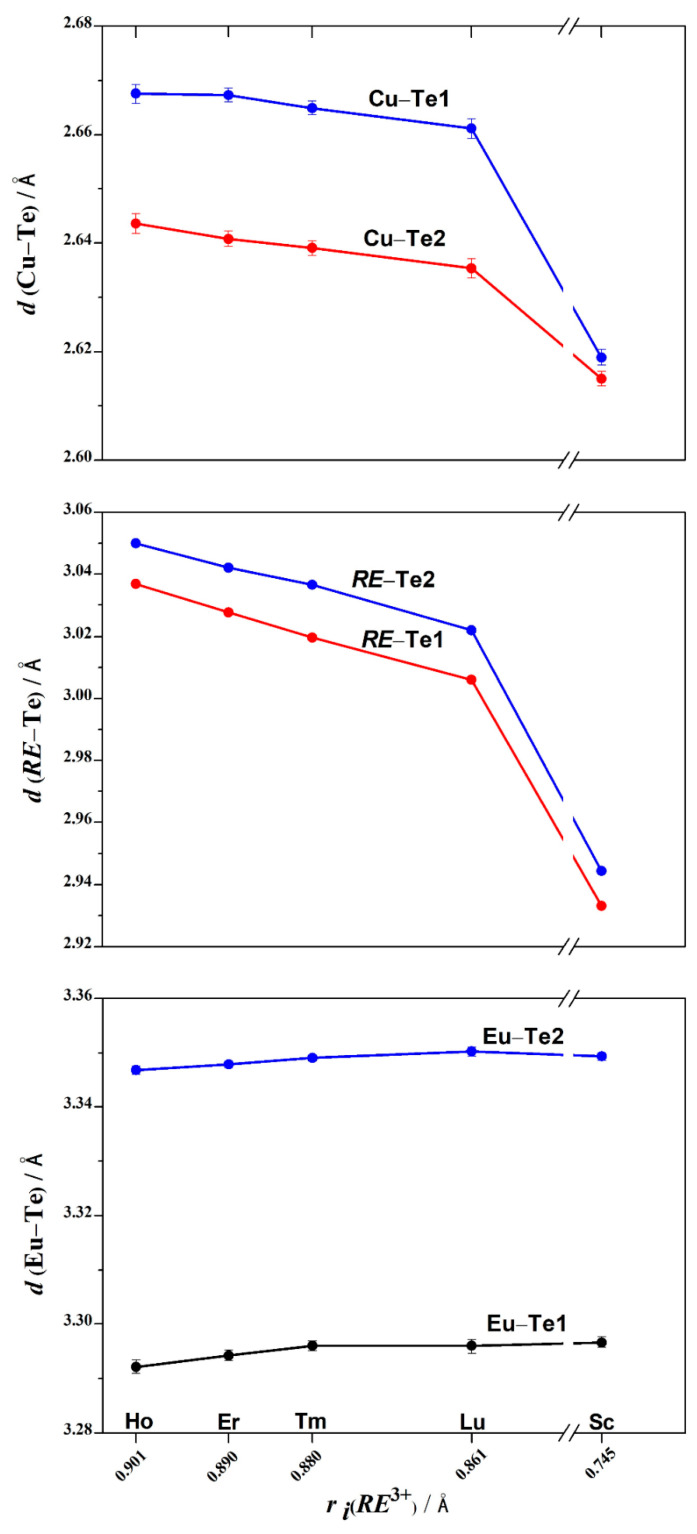
The distance *d*(*M*–Te) in the structures of Eu*RE*CuTe_3_ compounds with *M* = Eu, Cu, *RE* (= Ho [this work], Er [19], Tm [this work], Lu [20], and Sc [this work]).

In the structures of Eu*RE*CuTe_3_ (*RE* = Ho, Tm, Sc), four distances *d*(Cu–Te) are shorter than the theoretical value of 2.81 Å [32] by 5–7%, while the fifth and sixth distances (4.293–4.086 Å) are longer by 45–52% (Figure S1). The typical coordination polyhedron of copper in this structure is a tetrahedron (Figure 5 and Figure S1). The tetrahedra [CuTe_4_]^7−^ are linked by shared anions (Te1)^2−^ along the *a*-axis (Figure 5). As the rare-earth metal cation radius decreased in the quaternary tellurides, a decrease in the ionicity of the Cu–Te bond was observed in the tetrahedra, resulting in the bond lengths *d*(Cu–Te) changing from 2.644 to 2.615 Å and from 2.668 to 2.620 Å (Table S3, Figure 6).

**Figure 7:**
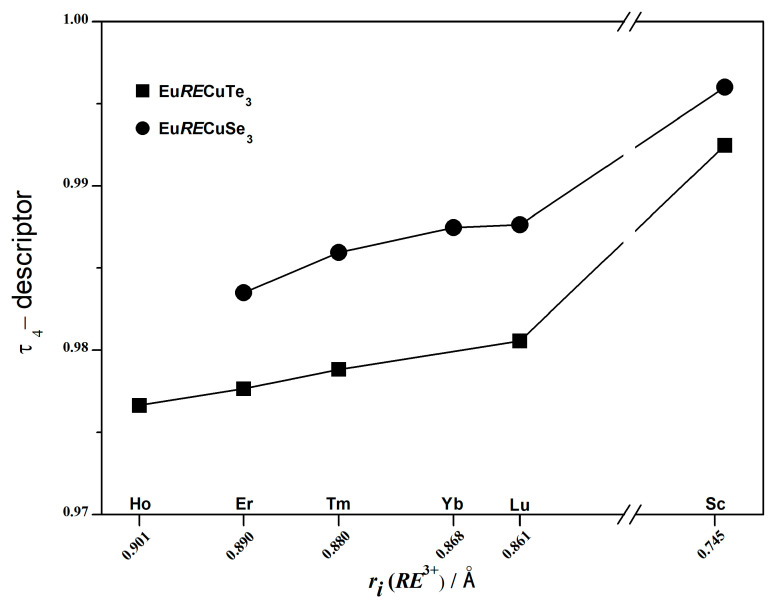
Calculated values of the τ_4_ descriptor for the polyhedra [Cu*Ch*_4_]^7−^ (*Ch* = Se, Te) in the structures of the compounds Eu*RE*CuTe_3_ (*RE* = Ho [this work], Er [19], Tm [this work], Yb [this work], Lu [20], Sc [this work]) and Eu*RE*CuSe_3_ (*RE* = Er [26], Tm–Lu [25], Sc [18]) in the space group *Cmcm*.

The values of the valence angles ∠(Te–Cu–Te), ranging from 104.4 to 111.0° (Table S3), deviate from the ideal tetrahedral angle by 1–4%. As the compound changed from EuHoCuTe_3_ to EuScCuTe_3_, this deviation decreases. The reduction in the distortion of [CuTe_4_]^7−^ in the series of compounds Eu*RE*CuTe_3_ (*RE* = Ho–Lu, Sc) is confirmed by the calculation of the τ_4_ descriptor [46], which increases in this series from 0.977 to 0.992 (Figure 7). Since the τ_4_ descriptor values for an ideal tetrahedron, trigonal pyramid, square pyramid, and ideal square are 1.00, 0.85, 0.64–0.07, and 0.00, respectively [46], in the structures of Eu*RE*CuTe_3_, a distortion of the copper coordination polyhedron from an ideal tetrahedron to a trigonal pyramid is observed at 2.3–0.8%. The scandium telluride possesses an almost ideal tetrahedral coordination environment. The degree of distortion of the tetrahedral [Cu*Ch*_4_]^7−^ polyhedra (*Ch* = Se, Te) in the tellurides Eu*RE*CuTe_3_ was found to be higher than in the selenides Eu*RE*CuSe_3_ (Figure 7).

In the structures of the Eu*RE*CuTe_3_ compounds in the space group *Cmcm*, the distances *d*(*RE*–Te) (Table S3) deviate from theoretical values (2.96 Å (EuScCuTe_3_)–3.11 Å (EuHoCuTe_3_) [32]) by 1–2%. The coordination polyhedra of *RE*^3+^ in the structures of Eu*RE*CuTe_3_ (*RE* = Ho, Tm, Sc) are octahedra, which show distortions. The valence angles ∠(Te–*RE*–Te), ranging from 86.3 to 93.7° (Table S3), deviate from the ideal octahedral angle by 4%. The octahedral units [*RE*Te_6_]^9−^ are connected to each other through (Te1)^2−^ anions along the *c*-axis and through (Te2)^2−^ anions along the *a*-axis (Figure 5). The coordination polyhedra [*RE*Te_6_]^9−^ and [CuTe_4_]^7−^ share common anions (Se1)^2−^ and (Se2)^2−^ and form two-dimensional layers in the *ac*-plane. In the octahedron, as the radius of *RE*^3+^ decreases, there is a reduction in the bond lengths *d*(*RE*–Te) from 3.0368 to 2.9334 Å and from 3.0501 to 2.9449 Å (Table S3, Figure 6), leading to a crystal-chemical compression of the two-dimensional layers [*RE*CuTe_3_]^2−^.

The anions (Te2)^2−^ and (Te1)^2−^ form trigonal prisms [EuTe_6_]^10−^ around the Eu^2+^ cations (Figure S1) connected to each other along the *a*-axis. The length of the four bonds *d*(Eu–Te2) in the structures range from 3.3468 to 3.3491 Å, while the lengths of the other two bonds *d*(Eu–Te1) are between 3.292 and 3.2961 Å (Figure 6, Table S3). Six distances *d*(Eu–Te) deviate from the theoretical value of 3.38 Å [32] by 2.5–2.6%, while the seventh and eighth distances (3.877–3.706 Å) are longer by 9.6–14.7% (Figure S1). The sums of valence efforts for the compounds Eu*RE*CuTe_3_ (*RE* = Ho–Lu and Sc) taking coordination into account are Eu (1.63–1.65), *RE* (2.66–3.09), and Cu (1.33–1.57) (Table S5).

The crystal structure of compounds Eu*RE*CuTe_3_ (*RE* = Ho–Lu, Sc) is formed by parallel two-dimensional layers in the *ac*-plane, consisting of octahedra and tetrahedra, which are separated by one-dimensional chains of trigonal prisms (Figure 5).

### 3.2. Band Structure

**Figure 8:**
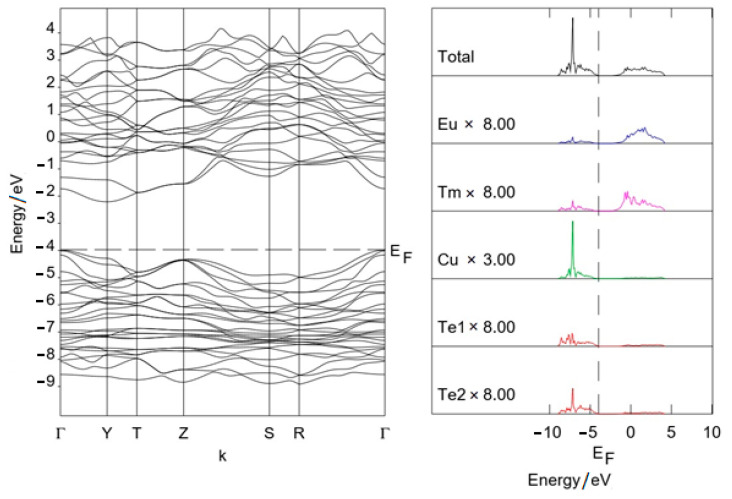
Band structure of EuTmCuTe_3_.

**Figure 9:**
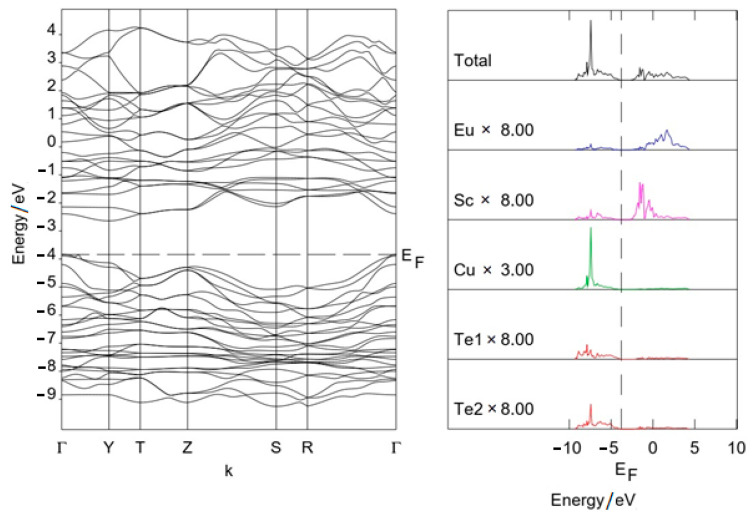
Band structure of EuScCuTe_3_.

The path in the Brillouin zone of the space group *Cmcm* is Г(0,0,0), Y(^1^/_2_,^1^/_2_,0), T(^1^/_2_,^1^/_2_,^1^/_2_), Z(0,0,^1^/_2_), S (0,^1^/_2_,0), R(0,^1^/_2_,^1^/_2_). The band structure (Figure 8 and Figure 9) does not include the 4f states of europium and *RE*^3+^, since they are replaced by pseudopotentials. As can be seen from the figures, copper and tellurium orbitals provide the main contribution to states near the top of the VB. Orbitals of *RE*^3+^ (Sc^3+^) and europium provide the main contribution to the bottom of the CB. Calculations predicted for Eu*RE*CuTe_3_ (*RE* = Ho, Tm, Yb, Lu) the indirect band gap Г–Y. The value of the band gap (1.7–1.8 eV) was a HOMO–LUMO estimation (Table 2). This value is close to experimental data for selenides (EuErCuSe_3_ 1.79 eV [26]). For EuScCuTe_3_, the calculation predicted a smaller gap, equal to 1.2 eV.

### 3.3. Elastic Constants and Elastic Modulus

**Figure 10:**
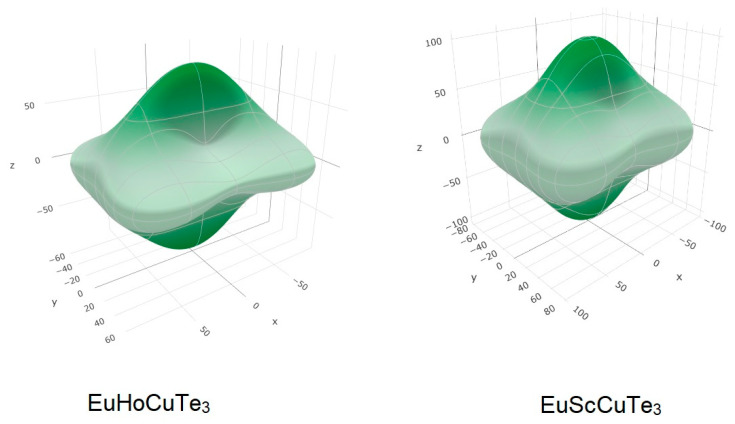
Young’s modulus (GPa). Dependence of the direction in the crystals EuHoCuTe_3_ and EuScCuTe_3._

The elastic constants and elastic modulus of the compound Eu*RE*CuTe_3_ (*RE* = Ho, Tm, Yb, Lu, Sc) are presented in Table 3. The table presents the bulk module (*B*), shear module (*G*), Young’s module (*Y*), and Poisson ratio. These are values for a polycrystal and were calculated by averaging the schemes of Voigt, Reuss, and Hill. The Voigt scheme assumes the uniformity of local strains. The Reuss scheme assumes the uniformity of local stresses. The Voigt scheme provides the upper bound, whereas the Reuss scheme provides the lower bound of value. The approximation of Hill is the average of Voigt and Reuss estimations [47]. The Voigt and Reuss estimates were found to be very different (Table 3), which indicates anisotropy of the elastic properties. The dependence of Young’s modulus on direction also illustrates the strong anisotropy of elastic properties (Figure 10).

(1) $$A^{U}=5\cdot \frac{G^{V}}{G^{R}}+\frac{B^{V}}{B^{R}}-6$$

We also calculated the universal elastic anisotropy index (1). The closer to zero this index is, the lower the anisotropy of elastic properties [48]. By the lanthanoid pressure, at the row Eu*RE*CuTe_3_ (*RE* = Ho–Lu), anisotropy decreases (Table 3).

(2) $$H_{V}=0.92{\left(\frac{G}{B}\right)}^{1.137}G^{0.708}$$

The empirical Formula (2) was used to calculate hardness. According to [49], the formula is based on correlations between Vickers hardness (*H_V_*) and the ratio of shear and bulk moduli. The parameters of the formula were determined from reproducing the hardness of more than forty compounds with ionic and covalent bonds [49]. The shear (*G*) and bulk (*B*) modulus in (2) is according to the Hill estimate.

### 3.4. Raman, IR, and Phonon Spectra

**Figure 11:**
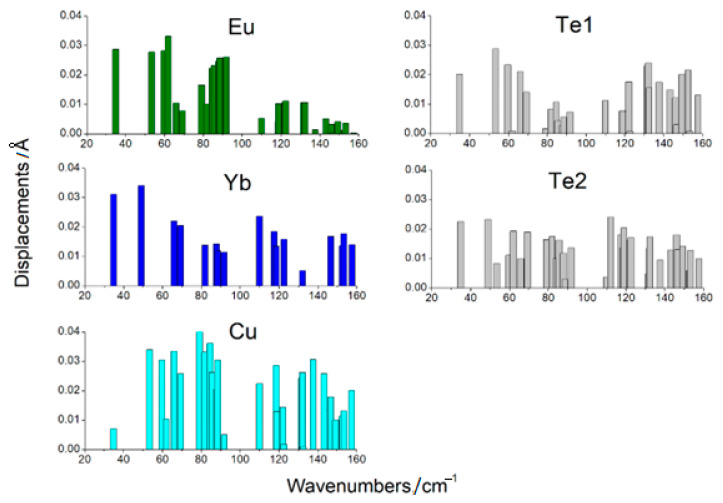
The values of ion displacements at phonon modes in EuYbCuTe_3_ (space group: *Cmcm*).

**Figure 12:**
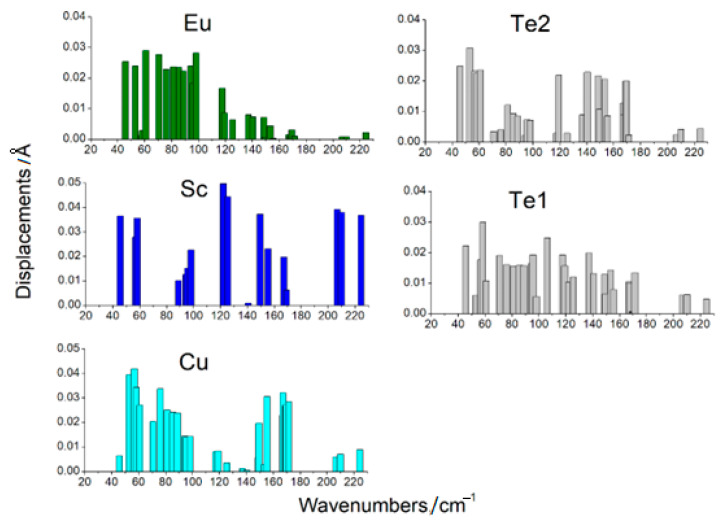
The values of ion displacements at phonon modes in EuScCuTe_3_ (space group: *Cmcm*).

From the DFT calculation, the wavenumbers and types of modes were determined (Table 4 and Table 5). The displacement vectors were obtained from the calculations also. This made it possible to evaluate the participation of each ion in a particular mode. The values of ion displacements characterized their participation in the mode (Figure 11 and Figure 12). According to calculations, the phonon spectrum of the crystals Eu*RE*CuTe_3_ (*RE* = Ho, Tm, Yb, Lu) at the gamma point lay in the frequency range up to 160 cm^−1^ (Table 4, Figure 11). In this frequency range, not only were light copper ions involved, but also telluride anions and *RE*^3+^ cations (Figure 11 and Figure 12). The phonon spectrum of the crystals EuScCuTe_3_ at the gamma point lay in the frequency range up to 230 cm^−1^ (Figure 12, Table 5). This corresponded to the fact that the mass of scandium is less than that of the other *RE*^3+^ cations. IR, Raman, and «silent» modes for all crystals are presented in Tables S6–S8.

A strong mixing of vibrations of structural units in crystals Eu*RE*CuTe_3_ can be noted. In all compounds, the europium ions participated in the frequency range up to ≈95 cm^−1^. Calculations predicted a gap in the phonon spectrum in the region ≈ 92–110 cm^−1^ for Eu*RE*CuTe_3_ (Figure 11). Note that a similar gap was practically absent in the crystal EuScCuTe_3_ (Figure 12).

**Figure 13:**
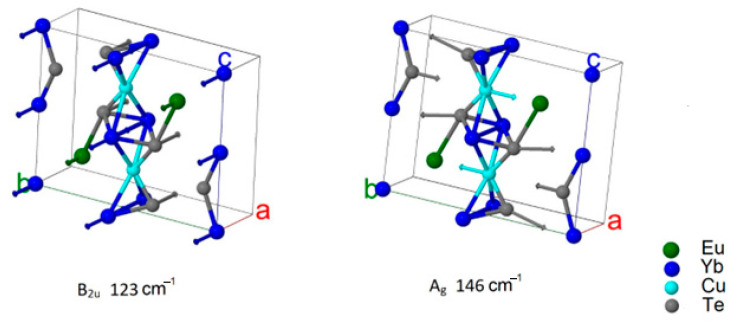
Ion displacements in IR and Raman modes with maximum intensity at EuYbCuTe_3_.

**Figure 14:**
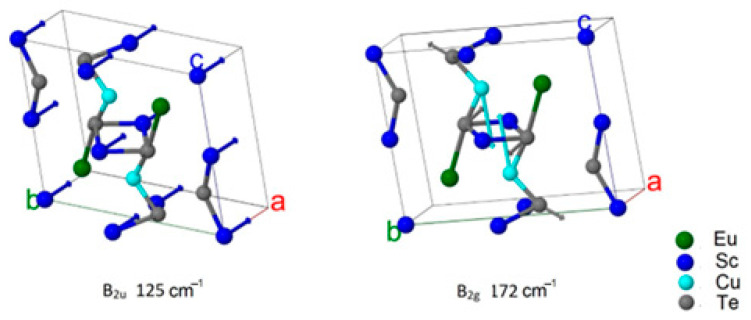
Ion displacements in IR and Raman modes with maximum intensity at EuScCuTe_3_.

**Figure 15:**
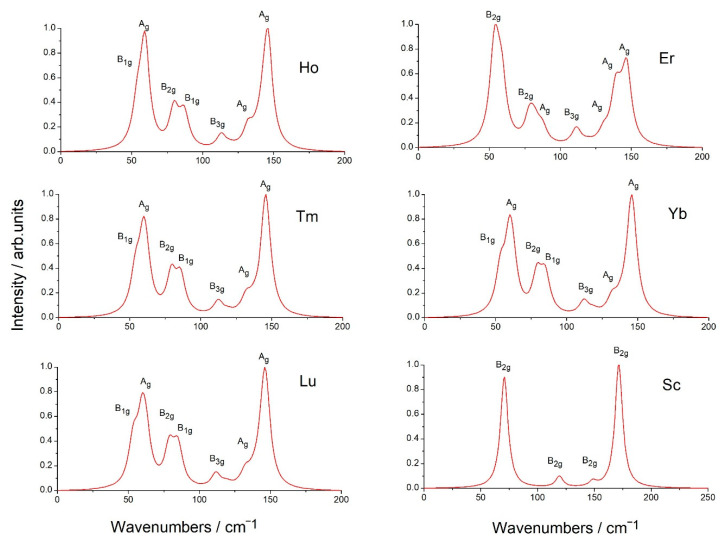
The simulated Raman spectra. The calculation was carried out for the exciting laser wavelength *λ* = 532 nm and *T* = 300 K. When modelling the Raman spectra based on the calculated wavenumbers and intensities, the functions pseudo-Voigt with dumping factor 8 cm^−1^ were used.

Calculations predicted that in crystals Eu*RE*CuTe_3_ (*RE* = Ho, Tm, Yb, Lu), the most intense Raman mode has a frequency of about 146 cm^−1^ (A_1g_), and the most intense infrared mode has a frequency of about 123 cm^−1^ (B_2u_). At the crystal EuScCuTe_3_, the most intense Raman mode has a frequency of about 172 cm^−1^ (B_2g_), and the most intense infrared modes has a frequency of about 125 cm^−1^ (B_2u_). These modes are illustrated in Figure 13 and Figure 14. The calculated Raman spectrum for all crystals is shown in Figure 15. The results of calculating the phonon spectrum can be useful for interpreting IR and Raman spectra of rare-earth metal tellurides Eu*RE*CuTe_3_. The IR and Raman spectra of Eu*RE*CuTe_3_ (*RE* = Ho, Tm, Lu) obtained from calculations are similar to the spectra of EuErCuTe_3_ [19]. It can be assumed that the experimental spectra of Eu*RE*CuTe_3_ (*RE* = Ho, Tm, Lu) will be similar to the previously presented experimental spectrum of EuErCuTe_3_ [19].

The largest ion displacement was 0.040 Å (Cu). In the case when the displacement was greater than or equal to 0.02 Å, the displacement is indicated by “S”. If the displacement did not exceed 0.01 Å, then the displacement is indicated by “W”. If the value of displacement was less than 0.005 Å, then the ion is not mentioned in the column “participants”.

The largest ion displacement is 0.05 Å (Sc). In the case when the displacement was greater than or equal to 0.02 Å, the displacement is indicated by “S”. If the displacement did not exceed 0.01 Å, then the displacement is indicated by “W”. If the value of displacement was less than 0.005 Å, then the ion is not mentioned in the column “participants”.

## 4. Conclusions

Single crystals of layered heterometallic tellurides Eu*RE*CuTe_3_ (*RE* = Ho, Tm, and Sc) were synthesized for the first time. The orthorhombic compounds crystallized in the space group *Cmcm*. With a decrease in the parameters and volume of the unit cell, the bond length *d*(*RE*–Te) occurred as the ionic radius of the rare-earth metal cation decreases (*RE*^3+^ = Ho^3+^, Er^3+^, Tm^3+^, Yb^3+^, Lu^3+^, Sc^3+^), leading to a crystal-chemical compression of the two-dimensional layers in the crystal structures of the Eu*RE*CuTe_3_ compounds. The patterns of changes in structural parameters were compared with the isostructural chalcogenides Eu*RE*Cu*Ch*_3_ (*Ch* = S, Se, Te) in the space group *Cmcm*. In the series of chalcogenides, the degree of distortion of the copper coordination polyhedra was found to be highest in the tellurides. Within the framework of the DFT approach, by using the hybrid functional, which takes into account non-local HF exchange, the crystal structure and IR, Raman, and “silent” modes were studied. Elastic tensor as well as elastic moduli and hardness were calculated. The theoretical calculations allow for the assignment of vibrational modes as well as revealing the involved ions that participated in these modes.

## Figures and Tables

**Table 1:** Main parameters of processing and refinement of the Eu*RE*CuTe_3_ (*RE* = Ho, Tm, Sc) samples.

	EuHoCuTe_3_	EuTmCuTe_3_	EuScCuTe_3_
Molecular weight (g/mol)	765.56	767.23	643.26
Space group	*Cmcm* (no. 63)		
Structure type	KZrCuS_3_		
*Z*	4		
*a* (Å)	4.3129(3)	4.3054(3)	4.2341(3)
*b* (Å)	14.3150(9)	14.3017(9)	14.1562(9)
*c* (Å)	11.2312(7)	11.1683(7)	10.8698(7)
*V* (Å^3^)	693.40(8)	687.68(8)	651.52(7)
*ρ*_cal_ (g/cm^3^)	7.311	7.410	6.558
*μ* (mm^−1^)	35.512	37.203	26.781
Reflections measured	6492	6639	6286
Reflections independent	481	478	451
Reflections with *F*_o_ > 4*σ*(*F*_o_)	0.030	0.021	0.031
*θ*_max_ (°)	27.44	27.48	27.53
*h, k, l* limits	−5 ≤ *h* ≤ 5, −18 ≤ *k* ≤ 18, −14 ≤ *l* ≤ 14		
*R*_int_, *R*_σ_	0.109, 0.047	0.069, 0.024	0.065, 0.026
*Refinement results*			
Number of refinement parameters	24		
*R*_1_ with *F*_o_ > 4*σ*(*F*_o_)	0.030	0.021	0.024
*wR*_2_	0.062	0.047	0.052
*Goof*	1.084	1.117	1.056
∆*ρ*_max_ (e/Å^3^)	1.857	1.431	1.503
∆*ρ*_min_ (e/Å^3^)	−1.783	−1.362	−1.467
Extinction coefficient, *ε*	0.00104(9)	0.00141(8)	0.0029(2)
CSD number	2261646	2261648	2354996

**Table 2:** Calculated band gaps of the tellurides Eu*RE*CuTe_3_ (*RE* = Ho–Lu and Sc) in eV.

Crystal	Indirect (Г–Y)	Crystal	Indirect (Г–Y)	Crystal	Indirect (Г–Y)
EuHoCuTe_3_	1.72	EuYbCuTe_3_	1.79	EuLuCuTe_3_	1.81
EuTmCuTe_3_	1.77	EuErCuTe_3_	1.75 [19]	EuScCuTe_3_	1.19

**Table 3:** Calculated elastic constants and modulus, as well as Vickers hardness (GPa) of the Eu*RE*CuTe_3_ series (*RE* = Ho, Tm, Yb, Lu, and Sc).

*RE*	C_11_	C_12_	C_13_	C_22_	C_23_	C_33_	C_44_	C_55_	C_66_	Averaging Scheme	*B*	*G*	*Y*	Poisson Ratio	*H_V_*	*A^U^*
Ho	121	52	40	94	47	113	9	31	45	Voigt	67	30	77	0.308	3.0	1.94
										Reuss	67	21	58	0.356		
										Hill	67	25	68	0.332		
Tm	121	52	40	96	48	114	11	31	45	Voigt	68	30	79	0.306	3.3	1.40
										Reuss	68	24	64	0.343		
										Hill	68	27	71	0.324		
Yb	122	52	40	96	47	114	11	31	45	Voigt	68	30	79	0.305	3.4	1.37
										Reuss	68	24	64	0.342		
										Hill	68	27	72	0.323		
Lu	122	52	40	97	48	115	12	31	45	Voigt	68	31	80	0.305	3.4	1.27
										Reuss	68	24	65	0.339		
										Hill	68	27	73	0.322		
Sc	127	59	42	103	50	127	18	33	45	Voigt	73	33	86	0.304	4.0	0.61
										Reuss	73	29	78	0.322		
										Hill	73	31	82	0.313		

**Table 4:** Phonons at the gamma point of EuYbCuTe_3_.

Frequency, cm^−1^	Type	IR		Raman		Involved Ions ^1^
		Active/Inactive	Intensity IR (km·mol^−1^)	Active/Inactive	Intensity Raman (Arbitrary Units)	
35	B_1u_	A	8.5	I		Eu ^S^, Yb ^S^, Cu ^W^, Te1 ^S^, Te2 ^S^
49	A_u_	I	0	I		Yb ^S^, Te2 ^S^
53	B_1g_	I	0	A	384	Eu ^S^, Cu ^S^, Te1 ^S^, Te2 ^W^
60	A_g_	I	0	A	576	Eu ^S^, Cu ^S^, Te1 ^S^, Te2
62	B_2g_	I	0	A	330	Eu ^S^, Cu ^W^, Te2
66	B_2u_	A	10.5	I		Eu, Yb ^S^, Cu ^S^, Te1 ^S^, Te2 ^W^
69	B_1u_	A	10.3	I		Eu ^W^, Yb ^S^, Cu ^S^, Te1, Te2
79	B_2g_	I	0	A	346	Eu, Cu ^S^, Te2
82	B_3u_	A	39.1	I		Eu, Yb, Cu ^S^, Te1 ^W^, Te2
85	B_1g_	I	0	A	218	Eu ^S^, Cu ^S^, Te1, Te2
86	A_g_	I	0	A	138	Eu ^S^, Cu ^S^, Te2
87.87	B_1u_	A	88.2	I		Eu ^S^, Yb, Cu ^S^, Te2
88.48	B_2u_	A	0.4	I		Eu ^S^, Yb, Cu ^S^, Te1 ^W^
92	B_3u_	A	128.3	I		Eu ^S^, Yb, Cu ^W^, Te1 ^W^, Te2
110	B_3u_	A	13.5	I		Eu^W^, Yb^S^, Cu^S^, Te1
112	B_3g_	I	0	A	133	Te2 ^S^
117	A_u_	I	0	I		Yb, Te2
118.56	B_1u_	A	390.4	I		Yb, Cu ^S^, Te1 ^W^, Te2
118.83	B_1g_	I	0	A	37	Eu, Cu, Te1 ^W^, Te2 ^S^
122	B_2g_	I	0	A	0.00	Eu, Cu, Te1, Te2
123	B_2u_	A	529.2	I		Eu, Yb, Te2
131.52	B_1g_	I	0	A	43	Eu, Cu ^S^, Te1 ^S^
132.12	B_3u_	A	78.8	I		Eu ^W^, Yb ^W^, Cu ^W^, Te1 ^S^, Te2
132.17	B_2u_	A	83.2	I		Eu ^W^, Cu ^S^, Te1 ^S^
132.34	A_g_	I	0	A	93	Eu, Te1, Te2
138	A_g_	I	0	A	62	Cu ^S^, Te1, Te2 ^W^
143	B_2g_	I	0	A	174	Eu ^W^, Cu ^S^, Te1, Te2
146	A_g_	I	0	A	1000	Cu, Te1, Te2
147	B_1u_	A	0.4	I		Yb, Cu, Te2
149	B_2g_	I	0	A	25	Cu, Te1 ^S^, Te2
153	B_1u_	A	111.2	I		Yb, Cu, Te1 ^S^, Te2 ^W^
153	B_3u_	A	170.4	I		Yb, Cu, Te2
158	B_3u_	A	10.2	I		Yb, Cu ^S^, Te1, Te2 ^W^

^1^ Superscripts “S” and “W” denote strong and weak ion displacements in the mode, respectively.

**Table 5:** Phonons at the gamma point of EuScCuTe_3_.

Frequency, cm^−1^	Type	IR		Raman		Involved Ions ^1^
		Active/Inactive	Intensity IR (km·mol^−1^)	Active/Inactive	Intensity Raman (Arbitrary Units)	
46	B_1u_	A	0	I		Eu ^S^, Sc ^S^, Cu ^W^, Te1 ^S^, Te2 ^S^
53	B_1g_	I	0	A	0.00	Eu ^S^, Cu ^S^, Te1 ^S^, Te2 ^W^
57	B_2u_	A	13.9	I		Sc ^S^, Cu ^S^, Te1 ^S^, Te2
58.23	B_1u_	A	20.3	I		Sc ^S^, Cu ^S^, Te1 ^S^, Te2 ^S^
58.30	A_u_	I	0	I		Sc ^S^, Te2 ^S^
61	A_g_	I	0	A	0.00	Eu ^S^, Cu ^S^, Te1 ^S^, Te2
71	B_2g_	I	0	A	896.00	Eu ^S^, Cu ^S^, Te2
76	B_2g_	I	0	A	0.31	Eu ^S^, Cu ^S^, Te2
81	B_1g_	I	0	A	0.00	Eu ^S^, Cu ^S^, Te1, Te2
86	A_g_	I	0	A	0.00	Eu ^S^, Cu ^S^, Te1 ^W^, Te2
89	B_3u_	A	142.4	I		Eu ^S^, Sc, Cu ^S^, Te1 ^W^, Te2
94	B_1u_	A	192.2	I		Eu ^S^, Sc, Cu, Te2
96	B_3u_	A	122.4	I		Eu, Sc, Cu, Te1 ^W^, Te2
98	B_2u_	A	0.01	I		Eu ^S^, Sc ^S^, Cu, Te1 ^W^, Te2 ^W^
106	B_3g_	I	0	A	0.00	Te2 ^S^
118	B_1g_	I	0	A	0.00	Eu, Cu ^W^, Te2
119	B_2g_	I	0	A	89.31	Eu ^W^, Cu ^W^, Te1 ^S^, Te2
122	A_u_	I	0	I		Sc ^S^, Te2
125	B_2u_	A	1388.7	I		Eu ^W^, Sc ^S^, Te2
137	A_g_	I	0	A	0.00	Eu ^W^, Te1 ^W^, Te2
141	B_3u_	A	30.2	I		Eu ^W^, Te1 ^S^, Te2
149	B_2g_	I	0	A	41.83	Eu ^W^, Cu ^W^, Te1 ^S^, Te2
150	B_1u_	A	1108.3	I		Sc ^S^, Cu, Te1, Te2 ^W^
153	A_g_	I	0	A	0.00	Te1 ^S^, Te2
155	B_3u_	A	5.3	I		Sc ^S^, Cu ^S^, Te1 ^W^, Te2 ^W^
167.18	B_1u_	A	96.4	I		Sc, Cu ^S^, Te1, Te2
167.26	A_g_	I	0	A	0.00	Cu ^S^, Te1 ^W^, Te2
169	B_2u_	A	131.6	I		Sc ^W^, Cu ^S^, Te1
170	B_1g_	I	0	A	0.00	Cu ^S^, Te1
172	B_2g_	I	0	A	1000.00	Cu ^S^, Te2
207	B_1u_	A	0.5	I		Sc ^S^, Cu ^W^, Te2 ^W^
210	B_3u_	A	33.6	I		Sc ^S^, Cu ^W^, Te2 ^W^
225	B_3u_	A	276.5	I		Sc ^S^, Cu ^W^

^1^ Superscripts “S” and “W” denote strong and weak ion displacements in the mode, respectively.

## Data Availability

The original contributions presented in the study are included in the article/Supplementary Material, further inquiries can be directed to the corresponding authors.

## Supplementary Materials

The following Supporting Information can be downloaded at: https://www.mdpi.com/article/10.3390/ma17143378/s1, Figure S1. Coordination polyhedra of [CuTe_4_]^7−^ (left), [EuTe_6_]^10−^ (right) in Eu*RE*CuTe_3_ (*RE* = Ho (this work), Er [19], Tm (this work), Lu [20], Sc (this work) from top to bottom). Table S1. Fractional atomic coordinates and isotropic or equivalent isotropic displacement parameters of the Eu*RE*CuTe_3_ (*RE* = Ho, Tm, Sc) samples. Table S2. Atomic displacement parameters (Å^2^) of the Eu*RE*CuTe_3_ (*RE* = Ho, Tm, Sc) samples. Table S3. Bond lengths (*d* /Å) and angles (∠ /°) in the crystal structures of the Eu*RE*CuTe_3_ representatives with *RE* = Ho, Tm and Sc. Table S4. Unit-cell parameters obtained by DFT methods. Table S5. Bond-valence calculation data for the Eu, *RE* and Cu cations in the Eu*RE*CuTe_3_ structure. Table S6. Calculated IR modes of the Eu*RE*CuTe_3_ representatives with *RE* = Ho, Tm, Yb, Lu and Sc. Table S7. Calculated Raman modes of the Eu*RE*CuTe_3_ representatives with *RE* = Ho, Tm, Yb, Lu and Sc. Table S8. Calculated “silent” modes of the Eu*RE*CuTe_3_ representatives with *RE* = Ho, Tm, Yb, Lu and Sc.
