# Supplementary material for: Syntheses and Patterns of Changes in Structural Parameters of the New Quaternary Tellurides EuRECuTe3 (RE = Ho, Tm, and Sc): Experiment and Theory
Source: Materials (Basel). 2024 Jul 9;17(14):3378. doi: 10.3390/ma17143378 (PMC11277929; doi:10.3390/ma17143378)
Supplement: Supplementary file 1 [file materials-17-03378-s001.zip › materials-3063351-supplementary.pdf]

Supplementary Materials

Syntheses and Patterns of Changes in Structural Parameters of the New Quaternary Tellurides Eu*RE*CuTe3 (*RE* = Ho, Tm, and Sc): Experiment and Theory

Anna V. Ruseikina [1,*], Maxim V. Grigoriev [1,2], Ralf J. C. Locke [2], Vladimir A. Chernyshev [3] and Thomas Schleid [2,*]

[1] Laboratory of Theory and Optimization of Chemical and Technological Processes, University of Tyumen, 625003 Tyumen, Russia; ma.v.grigorev@utmn.ru
[2] Institute for Inorganic Chemistry, University of Stuttgart, D-70569 Stuttgart, Germany; ralf.locke@iac.uni-stuttgart.de
[3] Institute of Natural Sciences and Mathematics, Ural Federal University named after the First President of Russia B.N. Yeltsin, 620002 Ekaterinburg, Russia; vchern@inbox.ru
* Correspondence: a.v.rusejkina@utmn.ru (A.V.R.); thomas.schleid@iac.uni-stuttgart.de (T.S.)

Table S1. Fractional atomic coordinates and isotropic or equivalent isotropic displacement parameters of the Eu*RE*CuTe3 (*RE* = Ho, Tm, Sc) samples.

**Atom**	*x*/*a*	*y*/*b*	*z*/*c*	**Atom**	*x*/*a*	*y*/*b*	*z*/*c*
Eu	0	0.75459(8)	1/4	Eu	0	0.75489(5)	1/4
Ho	0	0	0	Tm	0	0	0
Cu	0	0.47114(18)	1/4	Cu	0	0.47053(14)	1/4
Te1	0	0.08082(9)	1/4	Te1	0	0.08038(6)	1/4
Te2	0	0.35794(6)	0.06403(8)	Te2	0	0.35838(4)	0.06235(6)
Eu	0	0.75650(5)	1/4				
Sc	0	0	0				
Cu	0	0.46905(11)	1/4				
Te1	0	0.07804(6)	1/4				
Te2	0	0.36154(4)	0.05434(5)				

Table S2. Atomic displacement parameters (Å^2) of the Eu*RE*CuTe3 (*RE* = Ho, Tm, Sc) samples.

	U_{11}	U_{22}	U_{33}	U_{12}	U_{13}	U_{23}
			**EuHoCuTe3**			
Eu	0.0173(5)	0.0237(6)	0.0559(7)	0	0	0
Ho	0.0174(5)	0.0226(5)	0.0395(6)	0	0	–0.0057(4)
Cu	0.0266(13)	0.0282(15)	0.0359(14)	0	0	0
Te1	0.0182(7)	0.0192(7)	0.0304(7)	0	0	0
Te2	0.0172(5)	0.0200(5)	0.0364(6)	0	0	–0.0036(4)
			**EuTmCuTe3**			
Eu	0.0146(3)	0.0214(4)	0.0384(4)	0	0	0
Tm	0.0152(3)	0.0186(3)	0.0236(4)	0	0	–0.0017(2)
Cu	0.0253(9)	0.0248(10)	0.0288(9)	0	0	0
Te1	0.0154(4)	0.0168(5)	0.0201(5)	0	0	0
Te2	0.0149(3)	0.0166(3)	0.0241(3)	0	0	–0.0012(2)
			**EuScCuTe3**			
Eu	0.0145(3)	0.0213(4)	0.0270(4)	0	0	0.000
Sc	0.0164(11)	0.0150(11)	0.0172(11)	0	0	–0.0021(9)
Cu	0.0250(8)	0.0243(9)	0.0253(8)	0	0	0.000

Te1	0.0151(4)	0.0168(4)	0.0166(4)	0	0	0.000
Te2	0.0145(3)	0.0163(3)	0.0185(3)	0	0	0.0003(2)

Table S3. Bond lengths (*d* /Å) and angles (∠ /°) in the crystal structures of the Eu*RE*CuTe$_3$ representatives with *RE* = Ho, Tm and Sc.

**Bond lengths**					
		**EuHoCuTe$_3$**			
Eu–Te1^{i}	3.292(1)	Ho–Te1^{v}	3.0368(5)	Cu–Te2^{x}	2.644(2)
Eu–Te1^{ii}	3.292(1)	Ho–Te1	3.0368(5)	Cu–Te2	2.644(2)
Eu–Te2^{iii}	3.3468(8)	Ho–Te2^{vi}	3.0501(7)	Cu–Te1^{ii}	2.668(2)
Eu–Te2^{i}	3.3468(8)	Ho–Te2^{vii}	3.0501(7)	Cu–Te1^{i}	2.668(2)
Eu–Te2^{ii}	3.3468(8)	Ho–Te2^{viii}	3.0501(7)		
Eu–Te2^{iv}	3.3468(8)	Ho–Te2^{ix}	3.0501(7)		
		**EuTmCuTe$_3$**			
Eu–Te1^{i}	3.2960(9)	Tm–Te1	3.0195(4)	Cu–Te2	2.639(1)
Eu–Te1^{ii}	3.2960(9)	Tm–Te1^{v}	3.0194(4)	Cu–Te2^{x}	2.639(1)
Eu–Te2^{iii}	3.3491(6)	Tm–Te2^{vi}	3.0367(5)	Cu–Te1^{ii}	2.665(1)
Eu–Te2^{i}	3.3491(6)	Tm–Te2^{vii}	3.0367(5)	Cu–Te1^{i}	2.665(1)
Eu–Te2^{ii}	3.3491(6)	Tm–Te2^{viii}	3.0367(5)		
Eu–Te2^{iv}	3.3491(6)	Tm–Te2^{ix}	3.0367(5)		
		**EuScCuTe$_3$**			
Eu–Te1^{i}	3.2961(8)	Sc–Te1	2.9334(3)	Cu–Te2	2.615(1)
Eu–Te1^{ii}	3.2961(8)	Sc–Te1^{v}	2.9334(3)	Cu–Te2^{x}	2.615(1)
Eu–Te2^{iii}	3.3491(5)	Sc–Te2^{vi}	2.9449(4)	Cu–Te1^{ii}	2.620(1)
Eu–Te2^{i}	3.3491(5)	Sc–Te2^{vii}	2.9449(4)	Cu–Te1^{i}	2.620(1)
Eu–Te2^{ii}	3.3491(5)	Sc–Te2^{viii}	2.9449(4)		
Eu–Te2^{iv}	3.3491(5)	Sc–Te2^{ix}	2.9449(4)		
**Bond angles**					
		**EuHoCuTe$_3$**			
Te1^{i}–Eu–Te1^{ii}	81.84(4)	Te1^{v}–Ho–Te1	180.0	Te2–Cu–Te2^{x}	104.4(1)
Te1^{i}–Eu–Te2^{iii}	139.12(2)	Te1^{v}–Ho–Te2^{vi}	92.06(2)	Te2–Cu–Te1^{ii}	111.15(2)
Te1^{ii}–Eu–Te2^{iii}	84.95(2)	Te1–Ho–Te2^{vi}	87.94(2)	Te2^{x}–Cu–Te1^{ii}	111.15(2)
Te1^{i}–Eu–Te2^{i}	84.95(2)	Te1^{v}–Ho–Te2^{vii}	87.94(2)	Te2–Cu–Te1^{i}	111.15(2)
Te1^{ii}–Eu–Te2^{i}	139.12(2)	Te1–Ho–Te2^{vii}	92.06(2)	Te2^{x}–Cu–Te1^{i}	111.15(2)
Te2^{iii}–Eu–Te2^{i}	127.53(5)	Te2^{vi}–Ho–Te2^{vii}	180.00(3)	Te1^{ii}–Cu–Te1^{i}	107.9(1)
Te1^{i}–Eu–Te2^{iv}	84.95(2)	Te1^{v}–Ho–Te2^{viii}	87.94(2)		
Te1^{ii}–Eu–Te2^{iv}	139.12(2)	Te1–Ho–Te2^{viii}	92.06(2)		
Te2^{iii}–Eu–Te2^{iv}	80.23(2)	Te2^{vi}–Ho–Te2^{viii}	90.01(2)		
Te2^{i}–Eu–Te2^{iv}	77.23(3)	Te2^{vii}–Ho–Te2^{viii}	89.99(2)		
Te1^{i}–Eu–Te2^{ii}	139.12(2)	Te1^{v}–Ho–Te2^{ix}	92.06(2)		
Te1^{ii}–Eu–Te2^{ii}	84.95(2)	Te1–Ho–Te2^{ix}	87.94(2)		
Te2^{iii}–Eu–Te2^{ii}	77.23(3)	Te2^{vi}–Ho–Te2^{ix}	89.99(2)		
Te2^{i}–Eu–Te2^{ii}	80.23(2)	Te2^{vii}–Ho–Te2^{ix}	90.01(2)		
Te2^{iv}–Eu–Te2^{ii}	127.53(5)	Te2^{viii}–Ho–Te2^{ix}	180.00(3)		
		**EuTmCuTe$_3$**			
Te1^{i}–Eu–Te1^{ii}	81.56(3)	Te1^{v}–Tm–Te1	180.0	Te2–Cu–Te2^{x}	105.14(7)
Te1^{i}–Eu–Te2^{iii}	138.98(2)	Te1^{v}–Tm–Te2^{vi}	92.40(2)	Te2–Cu–Te1^{ii}	111.00(1)
Te1^{ii}–Eu–Te2^{iii}	85.11(1)	Te1–Tm–Te2^{vi}	87.60(2)	Te2^{x}–Cu–Te1^{ii}	111.00(1)
Te1^{i}–Eu–Te2^{i}	85.11(1)	Te1^{v}–Tm–Te2^{vii}	87.60(2)	Te2–Cu–Te1^{i}	111.00(1)
Te1^{ii}–Eu–Te2^{i}	138.98(2)	Te1–Tm–Te2^{vii}	92.40(2)	Te2^{x}–Cu–Te1^{i}	111.00(1)
Te2^{iii}–Eu–Te2^{i}	127.55(3)	Te2^{vi}–Tm–Te2^{vii}	180.00(3)	Te1^{ii}–Cu–Te1^{i}	107.76(8)

Te1^{i}–Eu–Te2^{iv}	85.11(1)	Te1^{v}–Tm–Te2^{viii}	87.60(2)		
Te1^{ii}–Eu–Te2^{iv}	138.98(2)	Te1–Tm–Te2^{viii}	92.40(2)		
Te2^{iii}–Eu–Te2^{iv}	80.00(2)	Te2^{vi}–Tm–Te2^{viii}	89.71(2)		
Te2^{i}–Eu–Te2^{iv}	77.47(2)	Te2^{vii}–Tm–Te2^{viii}	90.29(2)		
Te1^{i}–Eu–Te2^{ii}	138.98(2)	Te1^{v}–Tm–Te2^{ix}	92.40(2)		
Te1^{ii}–Eu–Te2^{ii}	85.11(1)	Te1–Tm–Te2^{ix}	87.60(2)		
Te2^{iii}–Eu–Te2^{ii}	77.47(2)	Te2^{vi}–Tm–Te2^{ix}	90.29(2)		
Te2^{i}–Eu–Te2^{ii}	80.00(2)	Te2^{vii}–Tm–Te2^{ix}	89.71(2)		
Te2^{iv}–Eu–Te2^{ii}	127.55(3)	Te2^{viii}–Tm–Te2^{ix}	180.00(2)		
		**EuScCuTe$_3$**			
Te1^{i}–Eu–Te1^{ii}	79.90 (3)	Te1^{v}–Sc–Te1	180.0	Te2–Cu–Te2^{x}	108.84(8)
Te1^{i}–Eu–Te2^{iii}	138.28(2)	Te1^{v}–Sc–Te2^{vi}	93.70(2)	Te2–Cu–Te1^{ii}	110.03(1)
Te1^{ii}–Eu–Te2^{iii}	86.25(1)	Te1–Sc–Te2^{vi}	86.30(2)	Te2^{x}–Cu–Te1^{ii}	110.03(1)
Te1^{i}–Eu–Te2^{i}	86.25(1)	Te1^{v}–Sc–Te2^{vii}	86.30(2)	Te2–Cu–Te1^{i}	110.03(1)
Te1^{ii}–Eu–Te2^{i}	138.28(2)	Te1–Sc–Te2^{vii}	93.70(2)	Te2^{x}–Cu–Te1^{i}	110.03(1)
Te2^{iii}–Eu–Te2^{i}	127.26(3)	Te2^{vi}–Sc–Te2^{vii}	180.0	Te1^{ii}–Cu–Te1^{i}	107.87(8)
Te1^{i}–Eu–Te2^{iv}	86.25(1)	Te1^{v}–Sc–Te2^{viii}	93.70(2)		
Te1^{ii}–Eu–Te2^{iv}	138.28(2)	Te1–Sc–Te2^{viii}	86.30(2)		
Te2^{iii}–Eu–Te2^{iv}	78.40(2)	Te2^{vi}–Sc–Te2^{viii}	91.94(2)		
Te2^{i}–Eu–Te2^{iv}	78.84(2)	Te2^{vii}–Sc–Te2^{viii}	88.06(2)		
Te1^{i}–Eu–Te2^{ii}	138.28(2)	Te1^{v}–Sc–Te2^{ix}	86.30(2)		
Te1^{ii}–Eu–Te2^{ii}	86.25(1)	Te1–Sc–Te2^{ix}	93.70(2)		
Te2^{iii}–Eu–Te2^{ii}	78.84(2)	Te2^{vi}–Sc–Te2^{ix}	88.06(2)		
Te2^{i}–Eu–Te2^{ii}	78.40(2)	Te2^{vii}–Sc–Te2^{ix}	91.94(2)		
Te2^{iv}–Eu–Te2^{ii}	127.26 (3)	Te2^{viii}–Sc–Te2^{ix}	180.0		

Symmetry codes: (i) $x-1/2$, $y+1/2$, z; (ii) $x+1/2$, $y+1/2$, z; (iii) $x+1/2$, $y+1/2$, $-z+1/2$; (iv) $x-1/2$, $y+1/2$, $-z+1/2$; (v) $-x$, $-y$, $-z$; (vi) $-x+1/2$, $-y+1/2$, $-z$; (vii) $x-1/2$, $y-1/2$, z; (viii) $x+1/2$, $y-1/2$, z; (ix) $-x-1/2$, $-y+1/2$, $-z$; (x) x, y, $-z+1/2$.

Table S4. Unit-cell parameters obtained by DFT methods.

Compound	Space Group	Structure Type	*a* (Å)	*b* (Å)	*c* (Å)
EuHoCuTe$_3$	*Cmcm*	KZrCuS$_3$	4.3435	14.2509	11.2627
EuTmCuTe$_3$	*Cmcm*	KZrCuS$_3$	4.3322	14.2552	11.2046
EuYbCuTe$_3$	*Cmcm*	KZrCuS$_3$	4.3267	14.2503	11.1792
EuLuCuTe$_3$	*Cmcm*	KZrCuS$_3$	4.3224	14.2393	11.1639
EuScCuTe$_3$	*Cmcm*	KZrCuS$_3$	4.2227	14.1817	10.7729

Table S5. Bond-valence calculation data for the Eu, *RE* and Cu cations in the Eu*RE*CuTe$_3$ structure.

Compound	Eu	*RE*	Cu
EuHoCuTe$_3$	1.65	3.09	1.41
EuTmCuTe$_3$	1.64	2.96	1.33
EuLuCuTe$_3$	1.63	2.92	1.44
EuScCuSe$_3$	1.64	2.66	1.56

Table S6. Calculated IR modes of the Eu*RE*CuTe$_3$ representatives with *RE* = Ho, Tm, Yb, Lu and Sc.

Type	Ho	Tm	Yb	Lu	Type	Sc
B_{1u}	32.4	34.6	34.9	35.4	B_{1u}	45.8
B_{2u}	67.0	65.9	66.0	64.2	B_{2u}	56.9
B_{1u}	70.9	70.3	69.3	68.8	B_{1u}	58.2
B_{3u}	81.3	81.6	81.9	82.0	B_{3u}	88.8
B_{1u}	87.3	87.9	87.9	88.1	B_{1u}	94.4
B_{2u}	90.9	89.7	88.5	88.8	B_{3u}	95.7
B_{3u}	91.6	91.8	91.7	91.7	B_{2u}	98.2
B_{3u}	111.9	110.9	110.0	109.6	B_{2u}	125.5
B_{1u}	119.2	119.0	118.6	118.6	B_{3u}	140.6
B_{2u}	123.0	122.5	122.5	121.6	B_{1u}	149.5
B_{3u}	131.6	131.6	132.1	132.5	B_{3u}	155.4
B_{2u}	132.0	131.9	132.2	132.7	B_{1u}	167.2
B_{1u}	148.4	148.0	146.7	146.7	B_{2u}	169.3
B_{1u}	154.4	153.5	152.5	152.1	B_{1u}	206.8
B_{3u}	155.9	154.7	153.3	152.8	B_{3u}	210.1
B_{3u}	159.6	158.3	157.5	157.4	B_{3u}	224.5

Table S7. Calculated Raman modes of the Eu*RE*CuTe$_3$ representatives with *RE* = Ho, Tm, Yb, Lu and Sc.

Type	Ho	Tm	Yb	Lu	Type	Sc
B_{1g}	54.0	54.1	53.4	53.6	B_{1g}	53.1
A_g	59.2	59.6	59.7	59.7	A_g	61.0
B_{2g}	59.3	61.6	61.9	62.6	B_{2g}	70.7
B_{2g}	79.8	79.4	79.1	78.9	B_{2g}	76.1
B_{1g}	87.6	85.8	84.5	84.5	B_{1g}	81.3
A_g	86.0	85.9	85.5	85.4	A_g	85.5
B_{3g}	113.2	112.3	112.1	111.7	B_{3g}	106.2
B_{1g}	119.5	119.2	118.8	119.1	B_{1g}	117.6
B_{2g}	123.1	122.9	122.0	121.4	B_{2g}	119.2
B_{1g}	131.9	131.7	131.5	132.1	A_g	137.2
A_g	131.8	132.2	132.3	132.4	B_{2g}	148.7
A_g	137.2	137.2	137.6	137.9	A_g	153.5
B_{2g}	142.5	144.3	143.2	143.9	A_g	167.3
A_g	145.8	146.0	146.0	146.3	B_{1g}	169.6
B_{2g}	149.5	149.6	149.3	149.1	B_{2g}	171.5

Table S8. Calculated "silent" modes of the Eu*RE*CuTe$_3$ representatives with *RE* = Ho, Tm, Yb, Lu and Sc.

Type	Ho	Tm	Yb	Lu	Sc
A_u	48.5	48.3	49.0	47.6	58.3
A_u	117.2	116.6	117.4	115.8	122.1

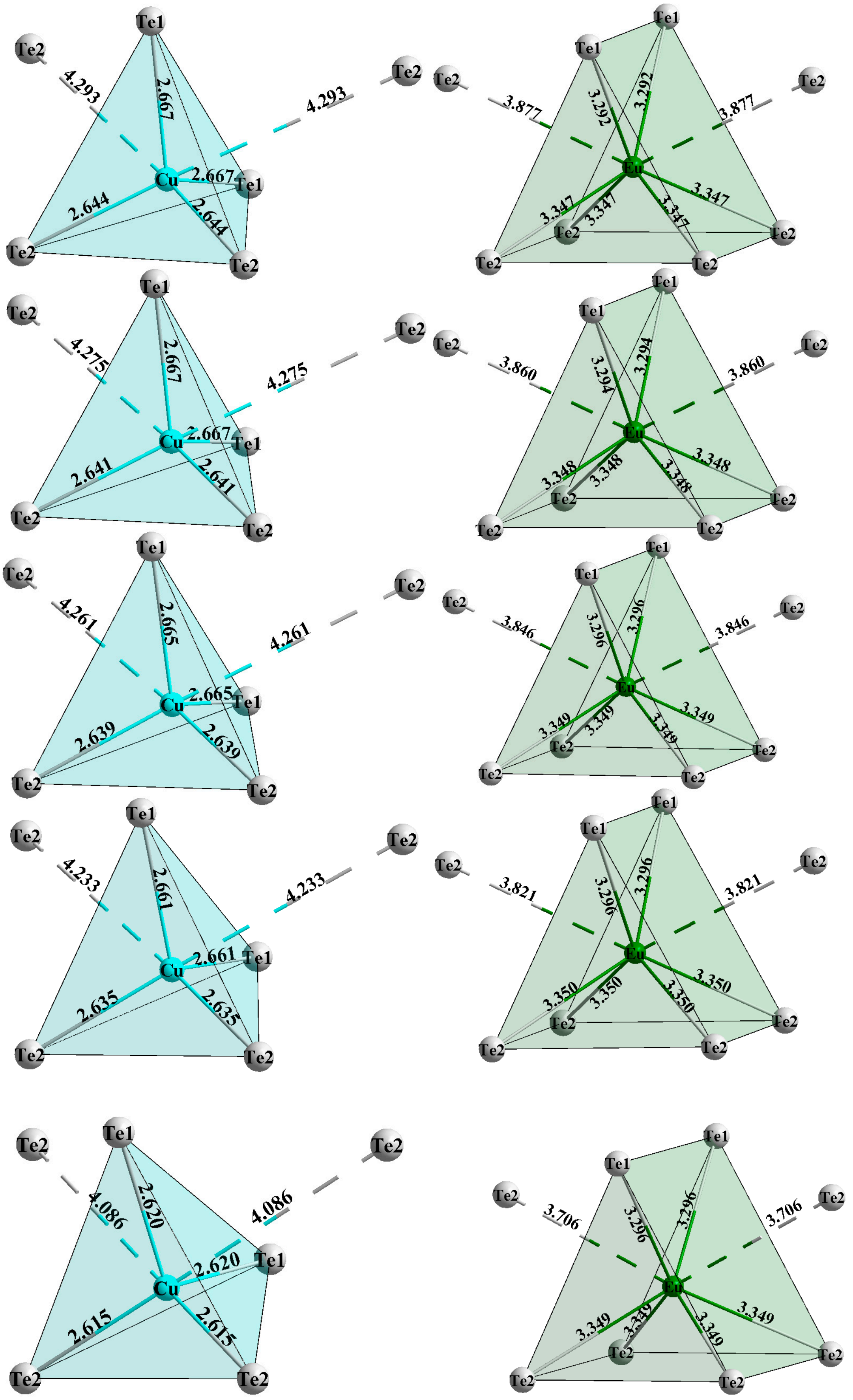

Figure S1. Coordination polyhedra of $[CuTe_4]^{7-}$ (left), $[EuTe_6]^{10-}$ (right) in Eu*RE*$CuTe_3$ (*RE* = Ho (this work), Er [23], Tm (this work), Lu [24], Sc (this work) from top to bottom).
